# The Association of Triglyceride to High-Density Lipoprotein Cholesterol Ratio with High-Risk Coronary Plaque Characteristics Determined by CT Angiography and Its Risk of Coronary Heart Disease

**DOI:** 10.3390/jcdd9100329

**Published:** 2022-09-28

**Authors:** Yuji Koide, Toru Miyoshi, Takahiro Nishihara, Mitsutaka Nakashima, Keishi Ichikawa, Takashi Miki, Kazuhiro Osawa, Hiroshi Ito

**Affiliations:** 1 Department of Cardiovascular Medicine, Okayama University Graduate School of Medicine, Dentistry and Pharmaceutical Sciences, Okayama 700-8558, Japan; 2 Department of Cardiovascular Medicine, Iwakuni Clinical Center, Iwakuni 740-8510, Japan; 3 Department of General Internal Medicine 3, Kawasaki Medical School General Medicine Center, Okayama 700-8505, Japan

**Keywords:** triglyceride, high density lipoprotein, coronary artery disease, computed tomography

## Abstract

The triglyceride to high-density lipoprotein cholesterol (TG/HDL-C) ratio is an independent risk index for cardiovascular events. This study aimed to evaluate the association between TG/HDL-C ratio and coronary plaque characteristics as seen on coronary computed tomography angiography (CCTA) and the corresponding increase in the likelihood of cardiovascular events. A total of 935 patients who underwent CCTA for suspected coronary artery disease (CAD) were included. High-risk plaques (HRP) were defined based on three characteristics: positive remodeling, low-density plaques, and spotty calcification. Significant stenosis was defined as luminal narrowing of >70%. Patients with a higher TG/HDL-C ratio showed significantly greater prevalence of HRP and significant stenosis than patients with low TG/HDL-C ratios (*p* < 0.01). Multivariate logistic analysis demonstrated that the TG/HDL-C ratio was significantly associated with the presence of HRP (*p* < 0.01) but not with significant coronary stenosis (*p* = 0.24). During the median follow-up period of 4.1 years, 26 cardiovascular events including cardiovascular death and acute coronary syndrome occurred. The highest TG/HDL-C tertile was associated with cardiovascular events, with the lowest TG/HDL-C tertile as the reference (hazard ratio, 3.75; 95% confidence interval, 1.04–13.50). A high TG/HDL-C ratio is associated with the presence of CCTA-verified HRP, which can lead to cardiovascular events in patients with suspected CAD.

## 1. Introduction

Abnormalities in serum lipids, including elevated low-density lipoprotein cholesterol (LDL-C), decreased high-density lipoprotein cholesterol (HDL-C), and increased blood triglyceride (TG) levels, are well-known risk factors for coronary artery disease (CAD) [1,2]. Although LDL-C is considered the most important risk factor, the TG/HDL-C ratio, known as the atherogenic index of plasma, provides additional risk stratification beyond that provided by LDL-C [3]. Previous studies have shown that the TG/HDL-C ratio is associated with CAD-related outcomes in both the general population [4,5,6] and high-risk patients with known CAD [7,8,9]. Although the relationship between the TG/HDL-C ratio and CAD is well known, its relationship with coronary plaque morphology remains unclear.

Coronary computed tomography angiography (CCTA) allows for the identification of high-risk coronary plaque characteristics and coronary atherosclerotic burden, both of which are involved in acute coronary syndrome [10,11,12]. An earlier study showed that the presence of low-attenuation plaques, positive remodeling, and spotty calcification are predictors of acute coronary syndrome [10]. Thus, the presence of high-risk plaque features and severe coronary stenosis determined using CCTA are useful for assessing the risk of CAD.

This study aimed to evaluate the association between TG/HDL-C ratio and coronary plaque characteristics that increase the likelihood of acute coronary events, and the association between TG/HDL-C ratio and the incidence of coronary events in patients with suspected CAD.

## 2. Materials and Methods

### 2.1. Study Population

**Figure 1:**
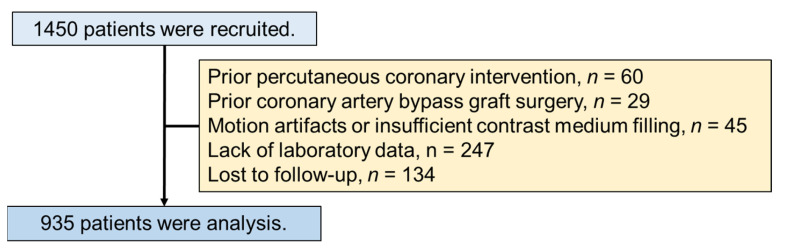
Flow chart of eligibility and exclusion of patients.

This study included patients with suspected coronary artery disease who underwent CCTA at Okayama University Hospital (Okayama, Japan) between August 2011 and August 2015. Exclusion criteria included prior percutaneous coronary intervention, prior coronary artery bypass graft surgery, severe heart failure (New York Heart Association classification > III), allergy to iodinated contrast agent, known severe renal failure (estimated glomerular filtration rate (eGFR) < 30 mL/min/1.73 m^2^), missing information regarding one or more traditional coronary artery risk factor(s) or laboratory data, poor image due to motion artifact, or inadequate contrast filling. As shown in Figure 1, 935 patients were included in this study. The study protocol was approved by the Institutional Ethics Committee on Human Research of Okayama University. The requirement for informed consent was waived owing to the low-risk nature of the study and the inability to obtain consent directly from all study subjects. The study conformed to the principles outlined in the Declaration of Helsinki.

### 2.2. Computed Tomography Image Acquisition 

Computed tomography (CT) scans were performed using a 128-slice CT scanner (SOMATOM Definition Flash; Siemens Medical Solutions, Erlangen, Germany) as described previously [13]. The initial bolus of contrast agent (Omnipaque 350; Daiichi Sankyo, Tokyo, Japan) was calculated as body weight × 0.07 mL and injected over 10 s. A CT acquisition protocol using a test bolus was carried out at the level of the ascending aorta after administration of 10 mL of the contrast medium (Omnipaque 350; Daiichi Sankyo, Tokyo, Japan), then 15 mL of physiological (0.9%) saline. Low-dose images were obtained every 1 s. The delay before formal imaging was calculated as the time to peak enhancement in the ascending aorta plus 3 s to ensure that enhancement of the contrast agent was 12 s, and was followed by a second bolus of the contrast agent that had been diluted 1:1 with physiologic saline for an additional 8 s. Subsequently, the contrast agent was “chased” with a bolus of physiologic saline (20 mL). The flow rate for all injections was (body weight) × 0.07 mL/s. All patients arrived at the hospital 1 h before the scheduled CT scanning time, and those with a persistently high heart rate of > 60 beats per min (bpm) received oral metoprolol (20–40 mg). If the heart rate did not sufficiently decrease to < 60 bpm before the scheduled CT scanning time, patients received intravenous landiolol hydrochloride (0.125 mg/kg) until the heart rate was < 60 bpm.

### 2.3. Coronary CT Angiography Analysis

The CT data were transferred to an office image analysis workstation. We used axial and curved multiplanar reformatted images to evaluate the morphology of coronary artery plaques using commercially available cardiac reconstruction software (Virtual Place; Raijin, AZE Inc., Tokyo, Japan) [14]. An experienced senior cardiologist evaluated the images on a per-segment basis with 16 segments examined as described previously using the segment model developed by the American Heart Association [15], thereby evaluating the presence and characteristics of coronary artery plaques on CT angiography. Coronary plaques were defined as structures > 1 mm^2^ within the coronary arteries that differed in density from that of the contrast-enhanced vessel lumen. Calcified plaques were defined as a minimum CT density of >130 Hounsfield units (HU), noncalcified plaques < 130 HU, or low-density plaques < 50 HU. Coronary artery remodeling was assessed by calculating the difference in vessel diameter at the plaque site compared to a reference site in a normal-appearing segment proximal to the lesion, with positive remodeling defined as an index of >1.05. Spotty calcification was defined as a calcium burden length <3/2 and width < 2/3 of the vessel diameter. We defined “high-risk plaque” as the presence of positive remodeling, low-density plaque, and spotty calcification, and we confirmed high-risk plaques when all these characteristics were present [16]. Significant coronary artery stenosis was defined as luminal obstruction of >70% of the diameter of the vessel.

### 2.4. Assessment of Other Risk Factors

Medical histories were investigated in all patients. Blood samples were drawn after an overnight fast at the central laboratory of Okayama University Hospital. Coronary risk factors were defined as follows: diabetes mellitus, defined as diagnosis in the past or self-reported history of, hemoglobin A1c level of >6.5% [17], or current or past use of hypoglycemic agents; dyslipidemia, defined as current or past use of lipid-lowering agents, LDL-C level ≥ 140 mg/dl, TG level ≥ 150 mg/dl, or HDL-C level < 40 mg/dl in a fasting blood sample; hypertension, defined as a resting blood pressure ≥140/90 mmHg or current or past use of antihypertensive agents [18]; smoking status, defined as regular smoking at the time of CT. Serum levels of TG and HDL-C were measured with the enzymatic color metric method. The TG/HDL-C ratio was calculated as TG (mg/dL) divided by HDL-C (mg/dL) [19]. 

### 2.5. Follow-Up Methods

Follow-up clinical information was obtained through a review of medical records or telephonic interviews by attending physicians. The primary outcome was defined as a composite of cardiovascular death and acute coronary syndrome. The secondary outcome was defined as a composite of cardiovascular death, acute coronary syndrome, or late coronary revascularization. Acute coronary syndrome was defined as both ST-segment elevation myocardial infarction and unstable angina or non-ST-segment elevation myocardial infarction. In all cases, acute coronary syndrome was recorded as an event only when the culprit was identified and urgent myocardial revascularization was performed. Late coronary revascularization was defined as nonurgent revascularization that occurred more than 90 days after index CCTA acquisition. Patients who underwent scheduled revascularization within 90 days after index CCTA were censored at the time of the first revascularization.

### 2.6. Statistical Analysis

Continuous variables were expressed as mean  ±  standard deviation or median with the interquartile range, depending on the Shapiro–Wilk test for normality. Dichotomous variables are expressed as numbers (proportions). Categorical data were compared using χ^2^ analysis or Fisher’s exact test among the groups. One-way analysis of variance was used to compare normally distributed continuous variables, and Bonferroni correction was used for post-hoc testing. The Kruskal–Wallis test was used to compare non-normally distributed continuous variables among the groups. Logistic analysis was performed to determine the odds ratio (OR) with 95% confidence intervals (95% CI) for TG/HDL-C ratio associated with HRP and significant stenosis. Cumulative survival estimates were calculated using the Kaplan–Meier method and compared using the log-rank test. To ascertain the association between TG/HDL-C ratio and the outcomes, we performed univariate and multivariate Cox regression analyses, and the results were reported as hazard ratio (HR) with 95% CI. To avoid overfitting in a multivariate Cox regression analysis, model 1 included TG/HDL ratio, age, male sex, LDL-C, hypertension, diabetes mellitus, current smoking, and the use of statins; Model 2 included HRP and significant stenosis in addition to the variables in model 1. All reported p-values were two-sided, and statistical significance was set at *p* < 0.05. Statistical analyses were performed using the SPSS statistical software (Version 28; IBM Corp., Armonk, NY, USA).

## 3. Results

### 3.1. Patient Characteristics

The baseline characteristics of the 935 patients according to TG/HDL-C ratio tertiles are summarized in Table 1. The mean age of all patients was 64 years, of which 55% were male, and 59%, 50%, and 33% of patients had hypertension, dyslipidemia, and diabetes mellitus, respectively. Patients were divided into tertiles based on their TG/HDL-C ratio (T1, n = 315, TG/HDL-C ratio < 1.56; T2, n =315, TG/HDL-C ratio > 1.57 and <2.66; T3, n = 305, TG/HDL-C ratio > 2.67). Among the three groups, the proportion of males, body mass index, prevalence of hypertension, dyslipidemia, diabetes mellitus, and current smoking increased significantly as the TG/HDL-C ratio increased. The levels of LDL-C, TG, and hemoglobin A1c increased, and HDL-C levels decreased as the TG/HDL-C ratio increased. With respect to medications, as the TG/HDL-C ratio increased, the proportion of patients using angiotensin-converting enzyme inhibitors or angiotensin-receptor blockers, and antidiabetic agents also increased.

### 3.2. CCTA Plaque Characteristics

Table 2 shows coronary artery plaque characteristics analysis with CCTA. HRP and significant stenosis were detected in 15% and 21% of patients, respectively. Among the three groups, as the TG/HDL-C ratio increased, the prevalence of calcified and noncalcified plaques, positive remodeling, low-density plaques, spotty calcification, high-risk plaques, and significant stenosis also increased. The Agatston score also increased significantly as the TG/HDL-C ratio increased.

Next, the association between TG/HDL-C ratio and HRP and significant stenosis was evaluated using logistic regression models (Table 3 and Table 4). In the univariate analysis, HRP was significantly associated with the TG/HDL-C ratio as well as with age, male sex, hypertension, dyslipidemia, diabetes mellitus, current smoking, and the use of statins, angiotensin-converting enzyme inhibitors or angiotensin-receptor blockers, calcium-channel blockers, and antidiabetic agents. In the multivariate analysis, the association between HRP and TG/HDL-C ratio remained significant (Table 3). In addition, in the univariate analysis, significant stenosis was significantly associated with the TG/HDL-C ratio as well as with age, male sex, hypertension, dyslipidemia, diabetes mellitus, and the use of statins, angiotensin-converting enzyme inhibitors or angiotensin-receptor blockers, calcium-channel blockers, and antidiabetic agents (Table 4). However, in the multivariate analysis, the association between significant stenosis and TG/HDL-C ratio was not significant. 

### 3.3. TG/HDL-C and Cardiovascular Events

**Figure 2:**
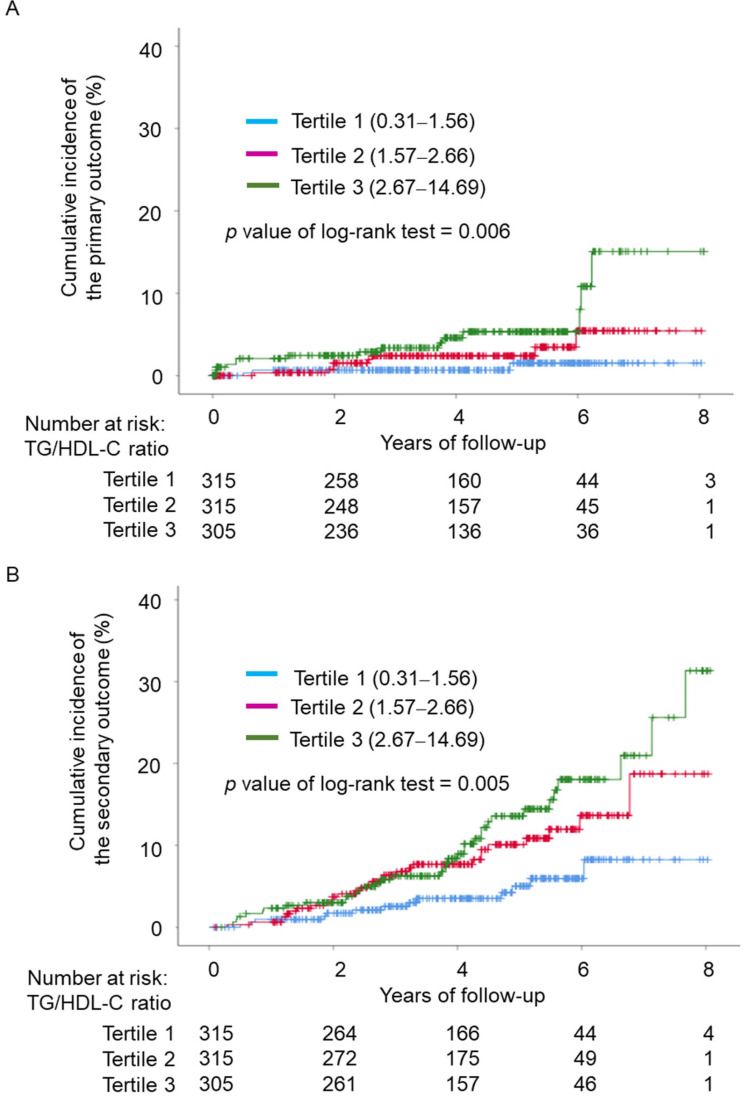
Kaplan–Meier plot of cumulative incidence of cardiovascular events by TG/HDL-C ratio tertiles. (**A**) Time to the primary outcome including cardiovascular death and acute coronary syndrome, according to the baseline TG/HDL-C ratio. (**B**) Time to the secondary outcome including cardiovascular death, acute coronary syndrome, or late coronary revascularization, according to the baseline TG/HDL-C ratio. TG, triglyceride; HDL-C, high-density lipoprotein cholesterol.

During the median follow-up period of 4.1 years, 78 cardiovascular events (4 cardiovascular deaths, 22 acute coronary syndrome, 51 late coronary revascularizations) occurred in the study group. The Kaplan–Meier plot demonstrated that the cumulative incidence rates of the primary outcome and the secondary outcome according to the TG/HDL-C ratio were significantly higher in the highest tertile group (TG/HDL-C ≥ 2.67) than in the first tertile group (TG/HDL-C ≤ 1.56) (P-values for trend = 0.006 and 0.005, respectively) (Figure 2A,B).

Table 5 shows the association between TG/HDL-C ratio and the primary outcome including cardiovascular death and acute coronary syndrome. Univariate Cox analysis showed that the highest tertile group was associated with the primary outcome, with the lowest tertile as the reference (*p* = 0.007). HRP (*p* = 0.011) and significant stenosis (*p* = 0.002) were also significant risk factors for the primary outcome. In multivariate Cox model 1, including the TG/HDL-C ratio and clinical variables, the highest group was significantly associated with the primary outcome (*p* = 0.043). Furthermore, in the multivariate Cox model 2, including HRP and significant stenosis in addition to model 1, the highest tertile group was not significant for the primary outcome (*p* = 0.052).

Table 6 shows the association between TG/HDL-C ratio and the secondary outcome including cardiovascular death, acute coronary syndrome, or late coronary revascularization. Univariate Cox analysis showed that the highest and middle tertile groups were associated with the primary outcome, with the lowest tertile as the reference (*p* = 0.002 and *p* < 0.023, respectively). HRP (*p* < 0.001) and significant stenosis (*p* < 0.001) were also significant risk factors for the secondary outcome. In multivariate Cox model 1, including the TG/HDL-C ratio and clinical variables, the highest group was significantly associated with the secondary outcome (*p* = 0.030). Furthermore, in the multivariate Cox model 2, including HRP and significant stenosis in addition to model 1, the highest tertile group was not significant for the secondary outcome (*p* = 0.064).

## 4. Discussion

The major finding of this study was that a high TG/HDL-C ratio was significantly associated with the presence of CCTA-verified HRP. To our knowledge, this is the first detailed study to evaluate the association between TG/HDL-C ratio and coronary plaque characteristics in patients with suspected CAD. Furthermore, the increased TG/HDL-C ratio was shown to be a significant predictor of adverse coronary events after adjustment for traditional risk factors, suggesting that the TG/HDL-C ratio is a potential biomarker for assessing CAD risk.

This study showed that an increased TG/HDL-C ratio, but not LDL-C, was associated with the presence of high-risk coronary plaques, which may predict future acute coronary syndrome [20]. In line with this study, we have reported that increased levels of oxidized LDL or HDL, but not the levels of LDL-C, were associated with the presence of high-risk coronary plaque in patients with suspected CAD [18,21]. Although LDL-C is considered the most important risk factor, emerging evidence suggests that the TG/HDL-C ratio provides additional risk stratification beyond that provided by LDL-C [3]. The pathophysiological implications of elevated TG and low HDL-C levels have been previously reported. High TG/HDL-C values are associated with metabolic syndrome, insulin resistance [8,22] and in general, a diabetic or pre-diabetic state [23]. Patients with high TG/HDL-C ratios have high values of remnant-C, which has been implicated in atherogenesis and inflammation [24]. TG-rich lipoproteins carry cholesterol in addition to TGs, and this, together with LDL-C, is considered the atherogenic agent that feeds the development of arterial wall plaques. In addition, elevated TG levels are associated with lower HDL-C levels and the formation of small dense LDL particles [25]. A recent study on intracoronary imaging using optical coherence tomography revealed that high levels of small dense LDL-C are associated with the presence of vulnerable plaques [26]. The TG/HDL-C ratio is positively correlated with insulin resistance [25]. Emerging data are linking TG/HDL-C ratio to metabolic syndrome, non-alcohol fatty liver disease, and diabetes mellitus [27,28,29]. Earlier studies have shown the prevalence of high-risk plaques detected by CCTA in patients with diabetes mellitus and metabolic syndrome [16,30,31]. Thus, cardiometabolic abnormalities reflecting the TG/HDL-C ratio may also explain the link between this marker and high-risk coronary plaques.

The relationship between the TG/HDL-C ratio and CAD-related outcomes has previously been described in the general population [4,5,6] and in high-risk patients with known CAD [7,8,9]. Cheng et al. reported that, in the general population, the highest tertile of TG/HDL ratio had almost 1.5-fold the prevalence of atherosclerotic cardiovascular events, with the lowest TG/HDL-C tertile as the reference [6]. Rohullah et al. reported that in high-risk patients, a TG/HDL-C ratio ≥ 2.5 was associated with almost a three-fold increase in cardiovascular events during a five-year follow-up, independent of traditional coronary risk factors and angiographic CAD severity [9]. In line with these studies, we demonstrated that the highest tertile of the TG/HDL-C ratio (> 2.66) had two-fold the prevalence of cardiovascular events in patients with suspected CAD after adjusting for traditional risk factors, with the lowest TG/HDL-C tertile as the reference. The TG/HDL-C ratio can easily be calculated based on commonly available parameters and yield strong prognostic significance in the general population as well as in high-risk patients with CAD risk factors.

Patients with a high TG/HDL-C ratio generally have other metabolic risk factors, including features of metabolic syndrome. Although LDL-C can be substantially reduced by statins, the first step to be taken in these individuals to increase HDL-C and decrease TG levels is lifestyle modification, such as physical activity, to reduce body weight [32]. Pharmacological interventions should be considered if the above aims have not been achieved. Fibrates are commonly used to decrease triglyceride levels, but triglyceride-lowering therapy with fibrates has not been shown to reduce the risk of cardiovascular diseases in large, randomized control trials [33]. Further studies are warranted to establish clear guidelines with regards to the management of patients with a high TG/HDL-C ratio in order to reduce cardiovascular events.

Current guideline for the Evaluation and Diagnosis of Chest from American Heart association stated that CCTA can visualize and help to diagnose the extent and severity of nonobstructive and obstructive CAD, as well as atherosclerotic plaque composition and high-risk features [34]. For intermediate–high-risk patients with stable chest pain and no known CAD, CCTA is effective for diagnosis of CAD, for risk stratification, and for guiding treatment decisions. Based on our findings, patients with suspected CAD and high TG/HDL-C ratio should be evaluated by CCTA. A randomized study showed that the use of CTA in addition to standard care in patients with stable chest pain resulted in a significantly lower rate of death from coronary heart disease [35]. The risk stratification with TG/HDL-C ratio in addition to traditional risk factors may contribute to the improvement of prognosis.

Our study had several limitations. First, this was a retrospective single-center study. Patient selection may have been biased, and a prospective study is preferable. Second, we included only Asian patients with suspected CAD; therefore, the results cannot be applied to other ethnic groups and the general population. Third, information on the changes in medications after CCTA was not available. The intensification of lipid-lowering therapy during follow-up may weaken the association between the TG/HDL-C ratio and cardiovascular events. Fourth, the patient cohort was treated about 10 years ago. The implementation of the present study into current clinical practice needs to be cautious because the guidelines on primary prevention of cardiovascular disease has been changed. Fifth, information on cardiovascular events was obtained through a review of medical records or telephonic interviews retrospectively. The follow-up is not standardized and outcomes could be missed. In addition, high number of patients were excluded due to loss in follow-up. This may influence the results.

In conclusion, this study demonstrated that an increase in TG/HDL-C ratio was significantly associated with the presence of high-risk plaques determined by CCTA in patients with suspected stable CAD. Thus, the TG/HDL-C ratio may be a potential biomarker for assessing cardiovascular risk. Further studies are required to identify the best treatment method with which to mitigate the risk of patients with high TG/HDL-C ratio in order to protect against cardiovascular events.

## Figures and Tables

**Table 1:** Baseline characteristics of patients according to the TG/HDL ratio tertiles.

		TG/HDL Ratio			
	All	Tertile 1 (0.31–1.56)	Tertile 2 (1.57–2.66)	Tertile 3 (2.67–14.69)	*p* Value for Trend
n	935	315	315	305	
Age, years	64 ± 14	63 ± 15	64 ± 15	63 ± 12	0.436
Male	516 (55)	152 (48)	164 (52)	200 (55)	<0.001
BMI, kg/m^2^	23.8 ± 4.0	22.6 ± 3.9	23.7 ± 4.1	25.1 ± 3.5	<0.001
Hypertension	552 (59)	159 (21)	187 (59)	206 (67)	<0.001
Dyslipidemia	472 (50)	129 (41)	153 (48)	190 (62)	<0.001
Diabetes mellitus	306 (33)	84 (27)	103 (32)	123 (40)	0.001
Current smoking	211 (23)	53 (17)	68 (22)	90 (29)	<0.001
LDL-C, mg/dL	114 ± 31	107 ± 27	114 ± 32	121 ± 32	<0.001
TG, mg/dL	111 (2, 161)	73 (59, 88]	111 (96, 129]	207 ± 79	<0.001
HDL-C, mg/dL	58 ± 16	71 ± 16	56 ± 11	46 ± 10	<0.001
TG/HDL-C	1.98 (1.30, 3.16)	1.08 (0.83, 1.31)	2.00 (1.79, 2.27)	3.94 [3.18–5.25]	<0.001
HemoglobinA1c, %	6.3 ± 1.2	6.1 ± 1.0	6.3 ± 1.2	6.4 ± 1.3	<0.001
eGFR, mL/min/1.73 m^2^	69.7 ± 18	71.1 ± 19.0	68.4 ± 17.5	68.9 ± 16.7	0.154
Medications					
Statins	318 (34)	92 (29)	112 (35)	114 (37)	0.078
ACEIs or ARBs	355 (38)	102 (32)	127 (40)	126 (41)	0.042
CCBs	317 (34)	95 (30)	109 (35)	113 (37)	0.184
Antidiabetic agents	196 (23)	46 (15)	61 (20)	89 (29)	<0.001

Data are presented as number of patients (%) or mean ± standard deviation or median (interquartile range). BMI, body mass index; LDL-C, low-density lipoprotein cholesterol; HDL-C, high-density lipoprotein cholesterol; eGFR, estimated glomerular filtration rate; ACEI, angiotensin-converting enzyme inhibitor; ARB, angiotensin-receptor blocker; CCB, calcium-channel blocker.

**Table 2:** Numbers of plaques with various characteristics according to the TG/HDL ratio tertiles.

		TG/HDL Ratio			
	All	Tertile (0.31–1.56)	Tertile 2 (1.57–2.66)	Tertile 3 (2.67–14.69)	*p* Value for Trend
n	935	315	315	305	
Calcified plaque	608 (65)	201(56)	206 (65)	219 (72)	0.002
Non-calcified plaque	462 (49)	129 (41)	159 (51)	174 (57)	<0.001
Positive remodeling	373 (40)	95 (30)	132 (42)	146 (48)	<0.001
Low density plaque	277 (30)	66 (21)	100 (31)	111 (36)	<0.001
Spotty calcification	77 (24)	108 (34)	108 (34)	120 (39)	<0.001
High risk plaque	136 (15)	27 (9)	45 (14)	64 (21)	<0.001
Significant stenosis	193 (21)	53 (17)	63 (20)	77 (25)	0.033
Agatston score	20 (0, 245)	5 (0, 210)	33 (0, 286)	26 (0, 225)	0.016

Data are presented as the number of patients (%) or median [interquartile range]. TG, triglyceride; HDL-C, high-density lipoprotein cholesterol.

**Table 3:** Univariable and multivariable associates of high-risk plaques.

	Univariate Analysis		Multivariate Analysis	
	Odds Ratio (95% CI)	*p* Value	Odds Ratio (95% CI)	*p* Value
Log TG/HDL-C	2.009 (1.514–2.666)	<0.001	1.581 (1.150–2.173)	0.005
LDL-C	0.999 (0.994–1.005)	0.085	–	–
Age, per year	1.032 (1.016–1.048)	<0.001	1.029 (1.011–1.048)	0.002
Male	2.917 (1.925–4.422)	<0.001	2.797 (1.773–4.412)	<0.001
Hypertension	2.543 (1.673–3.876)	<0.001	1.129 (0.654–1.949)	0.664
Dyslipidemia	1.981 (1.358–2.892)	<0.001	1.472 (0.879–2.465)	0.142
Diabetes mellitus	1.998 (1.382–2.889)	<0.001	1.460 (0.803–2.655)	0.214
Current smoking	1.530 (1.020–2.296)	0.040	1.050 (0.655–1.657)	0.834
Statin	1.598 (1.103–2.313)	0.013	1.126 (0.682–1.857)	0.643
ACEIs or ARBs	2.534 (1.751–3.668)	<0.001	1.889 (1.192–2.992)	0.007
CCB	1.665 (1.151–2.409)	0.007	1.079 (0.695–1.675)	0.734
Antidiabetic agents	2.110 (1.414–3.147)	<0.001	1.032 (0.537–1.981)	0.925

CI, confidence interval; TG, triglyceride; HDL-C, high-density lipoprotein cholesterol; LDL-C, low-density lipoprotein cholesterol; ACEI, angiotensin-converting enzyme inhibitor; ARB, angiotensin receptor-blocker; CCB, calcium-channel blocker

**Table 4:** Univariable and multivariable associates of significant stenosis.

	Univariate Analysis		Multivariate Analysis	
	Odds Ratio (95% CI)	*p* Value	Odds Ratio (95% CI)	*p* Value
Log TG/HDL-C	1.551 (1.185–1.927)	<0.001	1.183 (0.893–1.568)	0.242
LDL-C	1.000 (0.995–1.00)	0.981	–	–
Age, per year	1.051 (1.035–1.067)	<0.001	1.055 (1.036–1.074)	<0.001
Male	2.829 (1.988–4.026)	<0.001	3.075 (2.064–4.580)	<0.001
Hypertension	2.051 (1.451–2.898)	<0.001	0.980 (0.617–1.557)	0.932
Dyslipidemia	2.214 (1.591–3.082)	<0.001	2.116 (1.340–3.342)	0.001
Diabetes mellitus	2.017 (1.459–2.789)	<0.001	1.390 (0.811–2.381)	0.231
Current smoking	1.399 (0.975–2.009)	0.069	1.043 (0.687–1.583)	0.843
Statins	1.628 (1.177–2.251)	0.003	0.898 (0.574–1.694)	0.639
ACEIs or ARBs	1.704 (1.237–2.346)	<0.001	1.128 (0.751–1.694)	0.562
CCB	1.778 (1.286–2.457)	<0.001	1.442 (0.969–2.147)	0.071
Antidiabetic agents	2.106 (1.472–3.013)	<0.001	1.182 (0.653–2.139)	0.581

CI, confidence interval; TG, triglyceride; HDL-C, high-density lipoprotein cholesterol; LDL-C, low-density lipoprotein cholesterol; ACEI, angiotensin-converting enzyme inhibitor; ARB, angiotensin-receptor blocker; CCB, calcium-channel blocker

**Table 5:** Univariable and multivariable associates of the primary outcome.

	Univariate Analysis		Multivariate Analysis: Model 1		Multivariate Analysis: Model 2	
	Hazard Ratio (95% CI)	*p* Value	Hazard Ratio (95% CI)	*p* Value	Hazard Ratio (95% CI)	*p* Value
TG/HDL-C ratio						
Tertile 1 (0.31–1.56)	reference	−	reference	−	reference	−
Tertile 2 (1.57–2.66)	2.733 (0.725–10.305)	0.138	2.339 (0.614–8.961)	0.213	2.308 (0.601–8.868)	0.223
Tertile 3 (2.67–14.69)	5.545 (1.605–19.159)	0.007	3.752 (1.043–13.501)	0.043	3.295 (0.994–12.003)	0.071
LDL-C, per 1 mg/dL	1.006 (0.994–1.018)	0.337	1.006 (0.993–1.020)	0.361	1.007 (0.994–1.020)	0.312
Age, per year	1.019 (0.987–1.053)	0.248	1.018 (0.981–1.056)	0.355	1.013 (0.976–1.051)	0.506
Male	2.349 (1.013–5.448)	0.047	1.452 (0.585–3.608)	0.421	1.212 (0.478–3.075)	0.686
Hypertension	3.039 (1.146–8.060)	0.026	1.961 (0.698–5.509)	0.201	1.822 (0.650–5.102)	0.254
Dyslipidemia	1.606 (0.728–3.539)	0.240	−	−	−	−
Diabetes mellitus	3.204 (1.452–7.073)	0.004	2.707 (1.178–6.233)	0.019	2.342 (1.009–5.440)	0.048
Current smoking	2.283 (1.033–5.045)	0.041	1.791 (0.749–4.278)	0.190	1.947 (0.817–4.639)	0.132
Statins	1.028 (0.458–2.306)	0.947	0.720 (0.309–1.679)	0.447	0.888 (0.387–2.037)	0.779
ACEIs or ARBs	2.731 (1.238–6.024)	0.013	−	−	−	−
CCBs	1.712 (0.782–3.745)	0.178	−	−	−	−
Antidiabetic agents	2.237 (1.013–4.938)	0.046	−	−	−	−
High-risk plaque	2.952 (1.283–6.794)	0.011	−	−	1.625 (0.645–4.091)	0.303
Significant stenosis	3.552 (1.566–8.058)	0.002	−	−	1.853 (0.731–4.678)	0.192

The primary outcome was defined as a composite of cardiovascular death and acute coronary syndrome. CI, confidence interval; TG, triglyceride; HDL-C, high-density lipoprotein cholesterol; LDL-C, low-density lipoprotein cholesterol; ACEI, angiotensin-converting enzyme inhibitor; ARB, angiotensin-receptor blocker; CCB, calcium-channel blocker.

**Table 6:** Univariable and multivariable associates of the secondary outcome.

	Univariate Analysis		Multivariate Analysis: Model 1		Multivariate Analysis: Model 2	
	Hazard Ratio (95% CI)	*p* Value	Hazard Ratio (95% CI)	*p* Value	Hazard Ratio (95% CI)	*p* Value
TG/HDL-C ratio						
Tertile 1 (0.31–1.56)	reference	−	reference	−	reference	−
Tertile 2 (1.57–2.66)	2.133 (1.109–4.103)	0.023	1.843 (0.950–3.578)	0.071	1.762 (0.906–3.427)	0.095
Tertile 3 (2.67–14.69)	2.754 (1.459–5.200)	0.002	2.085 (1.075–4.046)	0.030	1.884 (0.964–3.681)	0.064
LDL-C, per 1 mg/dL	1.003 (0.996–1.011)	0.356	1.005 (0.997–1.012)	0.257	1.004 (0.996–1.012)	0.319
Age, per year	1.032 (1.011–1.053)	0.003	1.033 (1.010–1.056)	0.004	1.027 (1.004–1.051)	0.021
Male	2.311 (1.411–2.786)	<0.001	1.858 (1.083–3.186)	0.024	1.539 (0.887–2.669)	0.125
Hypertension	1.740 (1.062–2.850)	0.028	1.211 (0.711–2.063)	0.480	1.118 (0.656–1.907)	0.682
Dyslipidemia	1.377 (0.874–2.167)	0.168	−	−	−	−
Diabetes mellitus	1.947 (1.249–3.036)	0.003	1.750 (1.099–2.789)	0.019	1.642 (1.027–2.625)	0.038
Current smoking	1.875 (1.176–2.990)	0.008	1.552 (0.945–2.547)	0.082	1.552 (0.945–2.555)	0.083
Statins	1.690 (1.225–2.331)	<0.001	0.837 (0.508–1.379)	0.485	0.798 (0.483–1.318)	0.378
ACEIs or ARBs	1.791 (1.149–2.793)	0.010	−	−	−	−
CCBs	1.856 (1.190–2.895)	0.006	−	−	−	−
Antidiabetic agents	1.454 (0.887–2.385)	0.138	−	−	−	−
High-risk plaque	3.033 (1.901–4.840)	<0.001	−	−	1.741 (1.041–2.910)	0.034
Significant stenosis	2.979 (1.897–4.676)	<0.001	−	−	1.706 (1.029–2.828)	0.038

The secondary outcome was defined as a composite of cardiovascular death, acute coronary syndrome, or late coronary revascularization. CI, confidence interval; TG, triglyceride; HDL-C, high-density lipoprotein cholesterol; LDL-C, low-density lipoprotein cholesterol; ACEI, angiotensin-converting enzyme inhibitor; ARB, angiotensin-receptor blocker; CCB, calcium-channel blocker.

## Data Availability

The data presented in this study are available upon request from the corresponding author. The data is not publicly available due to privacy concerns.
